# *catena*-Poly[[[aqua­[(2-hydroxy­phen­yl)acetato-κ^2^ *O*,*O*′]lead(II)]-μ_3_-[(2-hydroxy­phen­yl)acetato-κ^4^ *O*:*O*,*O*′:*O*′]] monohydrate]

**DOI:** 10.1107/S1600536810004162

**Published:** 2010-02-06

**Authors:** Jun-Xia Xiao, Xian-Ge Wu, Liang Qin

**Affiliations:** a School of Chemistry and Chemical Engineering, Zhaoqing University, Zhaoqing 526061, People’s Republic of China

## Abstract

In the title complex, {[Pb(C_8_H_7_O_3_)_2_(H_2_O)]·H_2_O}***_n_***, the Pb^II^ atom is seven-coordinated by six carboxyl­ate O atoms from four different 2-hydroxy­phenyl­acetate (2-dph) ligands and one water mol­ecule, displaying a hemidirected irregular geometry, with the empty side of the metal ion capped by a benzene ring forming a Pb⋯π contact [Pb⋯centroid distance = 3.342 (2) Å]. One 2-dph ligand functions in a bridging mode and connects Pb ions into a linear chain. The crystal packing is governed by intra- and inter­molecular O—H⋯O hydrogen bonds.

## Related literature

For general background to hydroxy­phenyl­acetate complexes, see: Liwporncharoenvong *et al.* (2002 ▶); Nie & Li (2009 ▶). For general background to hemi- and holodirected geometries of lead(II) complexes, see: Shimoni-Livny *et al.* (1998 ▶).
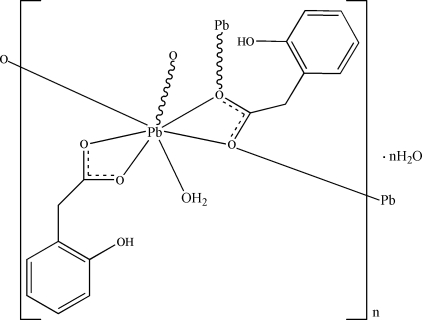


## Experimental

### 

#### Crystal data

[Pb(C_8_H_7_O_3_)_2_(H_2_O)]·H_2_O
*M* *_r_* = 545.50
Triclinic, 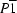

*a* = 7.4610 (15) Å
*b* = 10.721 (2) Å
*c* = 11.701 (2) Å
α = 109.72 (3)°
β = 90.10 (3)°
γ = 102.92 (3)°
*V* = 855.7 (3) Å^3^
*Z* = 2
Mo *K*α radiation
μ = 9.90 mm^−1^
*T* = 293 K
0.30 × 0.26 × 0.22 mm

#### Data collection

Rigaku/MSC Mercury CCD diffractometer
Absorption correction: multi-scan (*REQAB*; Jacobson, 1998 ▶) *T*_min_ = 0.075, *T*_max_ = 0.126
6821 measured reflections
3076 independent reflections
2951 reflections with *I* > 2σ(*I*)
*R*_int_ = 0.056

#### Refinement

*R*[*F* ^2^ > 2σ(*F* ^2^)] = 0.036
*wR*(*F* ^2^) = 0.084
*S* = 1.05
3076 reflections
228 parameters
6 restraints
H-atom parameters constrained
Δρ_max_ = 2.77 e Å^−3^
Δρ_min_ = −1.58 e Å^−3^

### 

Data collection: *CrystalStructure* (Rigaku/MSC, 2002 ▶); cell refinement: *CrystalStructure*; data reduction: *CrystalStructure*; program(s) used to solve structure: *SHELXS97* (Sheldrick, 2008 ▶); program(s) used to refine structure: *SHELXL97* (Sheldrick, 2008 ▶); molecular graphics: *ORTEPII* (Johnson, 1976 ▶) and *DIAMOND* (Brandenburg, 1999 ▶); software used to prepare material for publication: *SHELXL97*.

## Supplementary Material

Crystal structure: contains datablocks I, global. DOI: 10.1107/S1600536810004162/hy2276sup1.cif

Structure factors: contains datablocks I. DOI: 10.1107/S1600536810004162/hy2276Isup2.hkl

Additional supplementary materials:  crystallographic information; 3D view; checkCIF report

## Figures and Tables

**Table 1:** Selected bond lengths (Å)

Pb1—O1	2.701 (5)
Pb1—O2	2.527 (4)
Pb1—O3	2.453 (3)
Pb1—O3^i^	2.662 (4)
Pb1—O4	2.508 (4)
Pb1—O4^ii^	2.772 (4)
Pb1—O1*W*	2.687 (4)

Symmetry codes: (i) 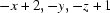; (ii) 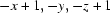.

**Table 2:** Hydrogen-bond geometry (Å, °)

*D*—H⋯*A*	*D*—H	H⋯*A*	*D*⋯*A*	*D*—H⋯*A*
O5—H5⋯O2*W*^iii^	0.82	1.80	2.618 (6)	173
O6—H6⋯O5^i^	0.82	1.93	2.698 (6)	156
O1*W*—H1*W*⋯O1^ii^	0.84	2.31	3.089 (6)	154
O1*W*—H2*W*⋯O2^i^	0.84	2.16	2.900 (6)	147
O2*W*—H3*W*⋯O1^iv^	0.84	1.88	2.715 (6)	172
O2*W*—H4*W*⋯O6^v^	0.84	2.05	2.788 (7)	146

Symmetry codes: (i) 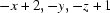; (ii) 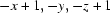; (iii) 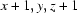; (iv) 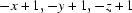; (v) 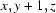.

**Fig. 1.**
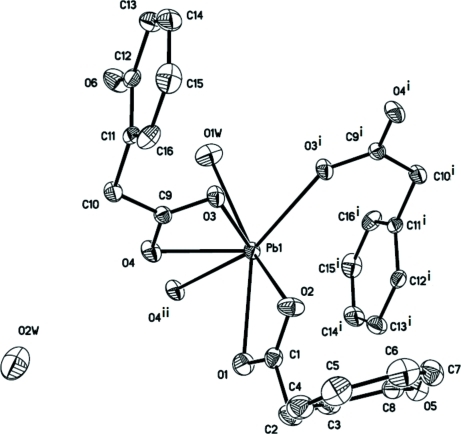
The asymmetric unit of the title compound, together with symmetry-related atoms to complete the coordination environment. Displacement ellipsoids are drawn at the 30% probability level. H atoms have been omitted for clarity. [Symmetry codes: (i) 2-x, -y, 1-z; (ii) 1-x, -y, 1-z.]

**Fig. 2.**
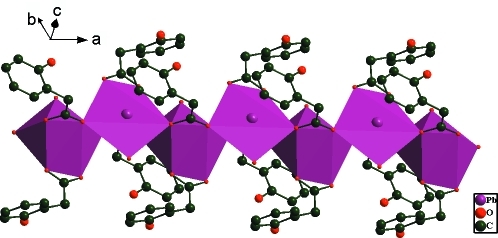
A polyhedral view of the Pb–carboxylate chain along the a axis. H atoms have been omitted for clarity.

## supplementary crystallographic information

### Comment

In the structural investigation of hydroxyphenylacetate complexes, it has been found that the hydroxyphenylacelic acid functions as a multidentate ligand (Liwporncharoenvong *et al.*, 2002; Nie & Li, 2009), with versatile binding and coordination modes. However, the structures of 2-hydroxyphenylacetate (2-dph) complexes have not been reported to date. In this paper, we report the crystal structure of the title compound, a new Pb complex obtained by the reaction of 2-Hdph and lead acetate in an alkaline aqueous solution.

As depicted in Fig. 1, the Pb^II^ atom is coordinated by six O atoms from four 2-dph ligands and one water molecule (Table 1). The coordination environment of the Pb^II^ atom can be described as a hemidirected irregular geometry, with the empty sapce around the meal ion filled by the stereochemically active 6 s^2^ electron pair (Shimoni-Livny *et al.*, 1998) and a Pb···π contact [Pb1···Cg1^i^ = 3.342 (2) Å, Cg1 is the centroid of the C11–C16 ring. Symmetry code: (i) 2-x, -y, 1-z]. The 2-dph ligands exhibit two types of coordination modes: one acts as bidentate chelating ligand; the other acts as a tetradentate chelate-bridging ligand to coordinate three Pb^II^ ions. The carboxylate groups of the tetradentate ligands connect Pb^II^ ions into a Pb–carboxylate linear chain, with Pb···Pb separations of 4.330 (2) and 4.381 (3) Å (Fig. 2). The crystal packing is governed by intra- and intermolecular O—H···O hydrogen bonding interactions involving the hydroxy and carboxylate groups of the 2-dph ligands, coordinated and uncoordinated water molecules (Table 2).

### Experimental

The title compound was prepared by the addition of a stoichiometric amount of lead acetate (1 mmol, 0.325 g) to a hot aqueous solution (25 ml) of 2-Hdph (1 mmol, 0.152 g). The pH value was then adjusted to 7.0 to 8.0 with NaOH (1 mmol, 0.04 g). The resulting solution was filtered, and colorless single crystals were obtained at room temperature over several days.

### Refinement

H atoms on C atoms and hydroxyl O atoms were placed at calculated positions and refined as riding atoms, with C—H = 0.93 (aromatic) and 0.97 (CH_2_) Å, O—H = 0.82 Å and with *U*_iso_(H) = 1.2*U*_eq_(C) or 1.5*U*_eq_(O). Water H atoms were tentatively located in difference Fourier maps and refined as riding, with distance restraints of O—H = 0.84 and H···H = 1.39 Å and with *U*_iso_(H) = 1.5*U*_eq_(O). The hightest peak in final difference map is located 0.94 Å from Pb1 and the deepest hole is located 0.97 Å from Pb1.

### Crystal data

**Table:**

[Pb(C_8_H_7_O_3_)_2_(H_2_O)]·H_2_O	*Z* = 2
*M**_r_* = 545.50	*F*(000) = 520
Triclinic, *P*1	*D*_x_ = 2.117 Mg m^−^^3^
Hall symbol: -P 1	Mo *K*α radiation, λ = 0.71073 Å
*a* = 7.4610 (15) Å	Cell parameters from 2895 reflections
*b* = 10.721 (2) Å	θ = 2.4–27.9°
*c* = 11.701 (2) Å	µ = 9.90 mm^−^^1^
α = 109.72 (3)°	*T* = 293 K
β = 90.10 (3)°	Block, colorless
γ = 102.92 (3)°	0.30 × 0.26 × 0.22 mm
*V* = 855.7 (3) Å^3^	

### Data collection

**Table:**

Rigaku/MSC Mercury CCD diffractometer	3076 independent reflections
Radiation source: fine-focus sealed tube	2951 reflections with *I* > 2σ(*I*)
graphite	*R*_int_ = 0.056
ω scans	θ_max_ = 25.2°, θ_min_ = 3.1°
Absorption correction: multi-scan (REQAB; Jacobson, 1998)	*h* = −8→8
*T*_min_ = 0.075, *T*_max_ = 0.126	*k* = −12→12
6821 measured reflections	*l* = −14→14

### Refinement

**Table:**

Refinement on *F*^2^	Primary atom site location: structure-invariant direct methods
Least-squares matrix: full	Secondary atom site location: difference Fourier map
*R*[*F*^2^ > 2σ(*F*^2^)] = 0.036	Hydrogen site location: inferred from neighbouring sites
*wR*(*F*^2^) = 0.084	H-atom parameters constrained
*S* = 1.05	*w* = 1/[σ^2^(*F*_o_^2^) + (0.0431*P*)^2^] where *P* = (*F*_o_^2^ + 2*F*_c_^2^)/3
3076 reflections	(Δ/σ)_max_ = 0.003
228 parameters	Δρ_max_ = 2.77 e Å^−^^3^
6 restraints	Δρ_min_ = −1.58 e Å^−^^3^

### Fractional atomic coordinates and isotropic or equivalent isotropic displacement parameters (Å^2^)

**Table:**

	*x*	*y*	*z*	*U*_iso_*/*U*_eq_	
Pb1	0.76187 (2)	0.029805 (17)	0.601979 (13)	0.02638 (11)	
O1	0.6798 (6)	0.2761 (5)	0.6889 (4)	0.0433 (10)	
O2	0.9615 (6)	0.2666 (4)	0.6423 (4)	0.0439 (10)	
O6	0.7995 (6)	−0.2407 (5)	0.1437 (5)	0.0487 (11)	
H6	0.8311	−0.3082	0.1465	0.073*	
O5	1.1923 (6)	0.5007 (4)	0.8890 (3)	0.0413 (9)	
H5	1.2721	0.5388	0.9460	0.062*	
C1	0.8449 (8)	0.3338 (6)	0.6845 (5)	0.0320 (12)	
C2	0.9012 (8)	0.4889 (6)	0.7318 (6)	0.0435 (14)	
H2A	0.8219	0.5223	0.6892	0.052*	
H2B	0.8802	0.5217	0.8175	0.052*	
C3	1.0981 (8)	0.5484 (5)	0.7178 (5)	0.0392 (13)	
C4	1.1459 (10)	0.6010 (6)	0.6257 (6)	0.0471 (15)	
H4	1.0530	0.6059	0.5753	0.056*	
C5	1.3273 (10)	0.6461 (7)	0.6070 (6)	0.0512 (17)	
H5A	1.3558	0.6801	0.5442	0.061*	
C6	1.4641 (10)	0.6410 (7)	0.6797 (6)	0.0531 (17)	
H6A	1.5865	0.6687	0.6652	0.064*	
C7	1.4221 (9)	0.5938 (6)	0.7770 (6)	0.0443 (14)	
H7	1.5162	0.5935	0.8289	0.053*	
C8	1.2403 (8)	0.5481 (5)	0.7949 (5)	0.0340 (12)	
C9	0.7658 (7)	0.0291 (5)	0.3582 (5)	0.0269 (11)	
C10	0.7743 (8)	0.0257 (7)	0.2278 (5)	0.0324 (12)	
H10A	0.6655	−0.0388	0.1794	0.039*	
H10B	0.7718	0.1151	0.2260	0.039*	
C11	0.9411 (7)	−0.0131 (6)	0.1715 (4)	0.0296 (12)	
C12	0.9491 (7)	−0.1495 (6)	0.1294 (4)	0.0300 (12)	
C13	1.1053 (8)	−0.1886 (7)	0.0786 (5)	0.0407 (14)	
H13	1.1097	−0.2799	0.0523	0.049*	
C14	1.2530 (9)	−0.0917 (8)	0.0675 (6)	0.0448 (16)	
H14	1.3572	−0.1176	0.0332	0.054*	
C15	1.2474 (9)	0.0435 (7)	0.1069 (5)	0.0478 (18)	
H15	1.3478	0.1088	0.0996	0.057*	
C16	1.0910 (9)	0.0828 (6)	0.1580 (5)	0.0390 (14)	
H16	1.0871	0.1741	0.1833	0.047*	
O3	0.9083 (5)	0.0283 (4)	0.4142 (3)	0.0339 (9)	
O4	0.6204 (5)	0.0363 (4)	0.4101 (3)	0.0366 (9)	
O1W	0.6888 (6)	−0.2332 (5)	0.4561 (4)	0.0531 (12)	
H2W	0.7696	−0.2339	0.4061	0.080*	
H1W	0.5860	−0.2720	0.4161	0.080*	
O2W	0.4261 (6)	0.6318 (6)	0.0817 (4)	0.0617 (14)	
H3W	0.3977	0.6548	0.1537	0.093*	
H4W	0.5291	0.6783	0.0746	0.093*	

### Atomic displacement parameters (Å^2^)

**Table:**

	*U*^11^	*U*^22^	*U*^33^	*U*^12^	*U*^13^	*U*^23^
Pb1	0.01845 (15)	0.03419 (16)	0.02621 (15)	0.00640 (11)	0.00500 (9)	0.00999 (11)
O1	0.029 (2)	0.046 (2)	0.049 (2)	0.005 (2)	0.0044 (18)	0.013 (2)
O2	0.031 (2)	0.034 (2)	0.063 (3)	0.0088 (19)	0.017 (2)	0.012 (2)
O6	0.031 (2)	0.041 (2)	0.075 (3)	0.006 (2)	0.004 (2)	0.022 (2)
O5	0.041 (2)	0.042 (2)	0.041 (2)	0.006 (2)	0.0043 (17)	0.0162 (19)
C1	0.026 (3)	0.038 (3)	0.030 (3)	0.006 (3)	0.002 (2)	0.010 (2)
C2	0.029 (3)	0.035 (3)	0.063 (4)	0.008 (3)	0.005 (3)	0.014 (3)
C3	0.034 (3)	0.025 (3)	0.055 (3)	0.005 (3)	0.003 (3)	0.010 (3)
C4	0.061 (4)	0.035 (3)	0.047 (3)	0.013 (3)	0.003 (3)	0.015 (3)
C5	0.055 (4)	0.043 (3)	0.055 (4)	0.002 (3)	0.010 (3)	0.023 (3)
C6	0.043 (4)	0.047 (4)	0.064 (4)	0.002 (3)	0.022 (3)	0.018 (3)
C7	0.031 (3)	0.044 (3)	0.056 (4)	0.005 (3)	0.002 (3)	0.018 (3)
C8	0.029 (3)	0.030 (3)	0.040 (3)	0.006 (2)	0.004 (2)	0.009 (2)
C9	0.022 (3)	0.028 (2)	0.029 (2)	0.006 (2)	0.003 (2)	0.009 (2)
C10	0.027 (3)	0.052 (3)	0.025 (3)	0.018 (3)	0.010 (2)	0.016 (2)
C11	0.029 (3)	0.040 (3)	0.017 (2)	0.014 (3)	0.0021 (19)	0.003 (2)
C12	0.026 (3)	0.036 (3)	0.022 (2)	0.006 (2)	0.0002 (19)	0.003 (2)
C13	0.039 (3)	0.048 (3)	0.039 (3)	0.021 (3)	0.008 (3)	0.013 (3)
C14	0.035 (3)	0.068 (4)	0.038 (3)	0.025 (3)	0.016 (3)	0.018 (3)
C15	0.033 (4)	0.061 (5)	0.048 (4)	0.000 (4)	0.007 (3)	0.024 (4)
C16	0.047 (4)	0.038 (3)	0.026 (2)	0.004 (3)	0.005 (2)	0.008 (2)
O3	0.0205 (19)	0.057 (2)	0.0277 (17)	0.0135 (18)	0.0076 (14)	0.0173 (17)
O4	0.0182 (19)	0.052 (2)	0.044 (2)	0.0106 (18)	0.0099 (16)	0.0217 (19)
O1W	0.046 (3)	0.045 (2)	0.057 (3)	0.000 (2)	0.019 (2)	0.011 (2)
O2W	0.036 (3)	0.092 (4)	0.044 (2)	0.018 (3)	0.007 (2)	0.005 (3)

### Geometric parameters (Å, °)

**Table:**

Pb1—O1	2.701 (5)	C6—H6A	0.9300
Pb1—O2	2.527 (4)	C7—C8	1.377 (8)
Pb1—O3	2.453 (3)	C7—H7	0.9300
Pb1—O3^i^	2.662 (4)	C9—O3	1.251 (6)
Pb1—O4	2.508 (4)	C9—O4	1.249 (6)
Pb1—O4^ii^	2.772 (4)	C9—C10	1.515 (7)
Pb1—O1W	2.687 (4)	C10—C11	1.485 (7)
O1—C1	1.259 (7)	C10—H10A	0.9700
O2—C1	1.249 (7)	C10—H10B	0.9700
O6—C12	1.362 (7)	C11—C12	1.392 (8)
O6—H6	0.8200	C11—C16	1.388 (8)
O5—C8	1.377 (7)	C12—C13	1.393 (8)
O5—H5	0.8200	C13—C14	1.375 (9)
C1—C2	1.521 (8)	C13—H13	0.9300
C2—C3	1.499 (8)	C14—C15	1.375 (9)
C2—H2A	0.9700	C14—H14	0.9300
C2—H2B	0.9700	C15—C16	1.396 (9)
C3—C4	1.389 (9)	C15—H15	0.9300
C3—C8	1.393 (8)	C16—H16	0.9300
C4—C5	1.376 (10)	O1W—H2W	0.8401
C4—H4	0.9300	O1W—H1W	0.8400
C5—C6	1.349 (10)	O2W—H3W	0.8365
C5—H5A	0.9300	O2W—H4W	0.8383
C6—C7	1.403 (9)		
			
O3—Pb1—O4	51.86 (12)	C5—C6—C7	120.2 (6)
O3—Pb1—O2	72.89 (14)	C5—C6—H6A	119.9
O4—Pb1—O2	89.65 (15)	C7—C6—H6A	119.9
O3—Pb1—O3^i^	64.41 (14)	C8—C7—C6	119.5 (6)
O4—Pb1—O3^i^	115.37 (11)	C8—C7—H7	120.2
O2—Pb1—O3^i^	80.89 (13)	C6—C7—H7	120.2
O3—Pb1—O1W	74.25 (14)	O5—C8—C7	121.6 (6)
O4—Pb1—O1W	77.54 (15)	O5—C8—C3	117.7 (5)
O2—Pb1—O1W	145.72 (13)	C7—C8—C3	120.7 (6)
O3^i^—Pb1—O1W	76.41 (14)	O3—C9—O4	120.4 (5)
O3—Pb1—O1	104.29 (14)	O3—C9—C10	118.9 (4)
O4—Pb1—O1	80.18 (14)	O4—C9—C10	120.6 (5)
O2—Pb1—O1	49.36 (13)	C11—C10—C9	113.5 (4)
O3^i^—Pb1—O1	128.80 (13)	C11—C10—H10A	108.9
O1—Pb1—O4^ii^	76.94 (13)	C9—C10—H10A	108.9
O2—Pb1—O4^ii^	125.05 (14)	C11—C10—H10B	108.9
O3—Pb1—O4^ii^	117.78 (12)	C9—C10—H10B	108.9
O3^i^—Pb1—O4^ii^	154.01 (12)	H10A—C10—H10B	107.7
O4—Pb1—O4^ii^	67.94 (11)	C12—C11—C16	118.2 (5)
O1W—Pb1—O4^ii^	79.49 (13)	C12—C11—C10	119.8 (5)
O1W—Pb1—O1	152.25 (15)	C16—C11—C10	122.1 (5)
C1—O1—Pb1	90.2 (4)	O6—C12—C11	116.6 (5)
C1—O2—Pb1	98.8 (3)	O6—C12—C13	122.3 (5)
C12—O6—H6	109.5	C11—C12—C13	121.1 (5)
C8—O5—H5	109.5	C14—C13—C12	119.7 (6)
O2—C1—O1	121.5 (5)	C14—C13—H13	120.1
O2—C1—C2	120.0 (5)	C12—C13—H13	120.1
O1—C1—C2	118.5 (5)	C15—C14—C13	120.3 (6)
C3—C2—C1	114.7 (5)	C15—C14—H14	119.9
C3—C2—H2A	108.6	C13—C14—H14	119.9
C1—C2—H2A	108.6	C14—C15—C16	120.0 (6)
C3—C2—H2B	108.6	C14—C15—H15	120.0
C1—C2—H2B	108.6	C16—C15—H15	120.0
H2A—C2—H2B	107.6	C11—C16—C15	120.7 (6)
C4—C3—C8	117.8 (6)	C11—C16—H16	119.6
C4—C3—C2	121.6 (6)	C15—C16—H16	119.6
C8—C3—C2	120.6 (6)	C9—O3—Pb1	95.1 (3)
C5—C4—C3	121.5 (7)	C9—O3—Pb1^i^	146.8 (3)
C5—C4—H4	119.2	Pb1—O3—Pb1^i^	115.59 (14)
C3—C4—H4	119.2	C9—O4—Pb1	92.5 (3)
C6—C5—C4	120.1 (6)	H2W—O1W—H1W	107.6
C6—C5—H5A	119.9	H3W—O2W—H4W	112.2
C4—C5—H5A	119.9		

Symmetry codes: (i) −*x*+2, −*y*, −*z*+1; (ii) −*x*+1, −*y*, −*z*+1.

### Hydrogen-bond geometry (Å, °)

**Table:**

*D*—H···*A*	*D*—H	H···*A*	*D*···*A*	*D*—H···*A*
O5—H5···O2W^iii^	0.82	1.80	2.618 (6)	173
O6—H6···O5^i^	0.82	1.93	2.698 (6)	156
O1W—H1W···O1^ii^	0.84	2.31	3.089 (6)	154
O1W—H2W···O2^i^	0.84	2.16	2.900 (6)	147
O2W—H3W···O1^iv^	0.84	1.88	2.715 (6)	172
O2W—H4W···O6^v^	0.84	2.05	2.788 (7)	146

Symmetry codes: (iii) *x*+1, *y*, *z*+1; (i) −*x*+2, −*y*, −*z*+1; (ii) −*x*+1, −*y*, −*z*+1; (iv) −*x*+1, −*y*+1, −*z*+1; (v) *x*, *y*+1, *z*.
